# Supplementary material for: Windborne migration amplifies insect-mediated pollination services
Source: eLife. 2022 Apr 13;11:e76230. doi: 10.7554/eLife.76230 (PMC9042232; doi:10.7554/eLife.76230)
Supplement: Supplementary file 1. — (a) Mass migration events of E. balteatus across the Bohai Strait observed by the searchlight trapping on BH Island during 2003–2018. (b) Collection information for sample in the study. (c) Pollen carrying rate of the migratory E. balteatus hoverflies across Bohai Sea during 2014–2018. (d) Quantitative PCR (qPCR) primers and conditions used in this study. (e) Key parameters within E. balteatus searchlight trapping on BH Island during 2003–2018. (f) The percentage of the total trajectories that ended in each region. (g) Results of analysis of molecular variance (AMOVA) test in different populations and regions of E.balteatus based on Cytb and18S-28S rRNA gene. (h) Comparative assessment of the degree of taxonomic identification obtained through either molecular or morphology-based approaches, for 46 different types of pollen grains dislocated from E. balteatus long-distance migrants collected on Beihuang Island (Bohai Sea, northeastern China). For each type of pollen grain, the highest level of taxonomic identification is indicated and contrasted between molecular and morphology-based approaches. [file elife-76230-supp1.docx]

**Supplementary File 1**

**Windborne migration amplifies insect-mediated pollination services**

**Supplementary file 1a.** Mass migration events of *E. balteatus* across the Bohai Strait observed by the searchlight trapping on BH Island during 2003–2018.

| **Year** | **Dates** | **Captured no.** | **Year** | **Dates** | **Captured no.** |
| --- | --- | --- | --- | --- | --- |
| 2003 | 3-Jun | 40 | 2010 | 30-May | 48 |
|  | 4-Jun | 50 |  | 1-Jun | 220 |
|  | 5-Jun | 45 |  | 6-Jun | 72 |
|  | 7-Jun | 136 |  | 14-Jun | 96 |
| 2004 | 15-May | 350 |  | 15-Jun | 66 |
|  | 29-May | 56 |  | 2-Sep | 1431 |
|  | 7-Jun | 51 |  | 5-Sep | 129 |
|  | 8-Oct | 60 |  | 11-Sep | 89 |
| 2005 | 26-May | 84 | 2011 | 18-May | 357 |
|  | 6-Jun | 49 |  | 6-Jun | 112 |
|  | 7-Sep | 270 |  | 22-Aug | 81 |
|  | 8-Sep | 96 |  | 23-Aug | 389 |
| 2006 | 19-May | 172 | 2012 | 23-May | 185 |
|  | 3-Jun | 265 |  | 27-May | 50 |
|  | 22-Aug | 183 |  | 5-Jun | 330 |
|  | 16-Sep | 120 |  | 20-Aug | 104 |
|  | 17-Sep | 68 |  | 9-Sep | 86 |
|  | 22-Sep | 128 | 2013 | 3-Jun | 129 |
|  | 30-Sep | 54 |  | 25-Aug | 61 |
|  | 3-Oct | 49 | 2014 | 21-May | 1018 |
| 2007 | 20-May | 79 |  | 29-May | 426 |
|  | 1-Sep | 185 |  | 29-Jun | 84 |
|  | 2-Sep | 86 | 2015 | 27-May | 59 |
|  | 6-Sep | 73 |  | 7-Jun | 67 |
|  | 10-Sep | 77 |  | 15-Jul | 184 |
| 2008 | 22-May | 105 |  | 24-Aug | 304 |
|  | 24-May | 248 |  | 27-Aug | 80 |
|  | 7-Jun | 134 |  | 4-Sep | 85 |
|  | 10-Jun | 116 | 2016 | 27-May | 65 |
|  | 1-Sep | 132 |  | 30-May | 49 |
|  | 2-Sep | 111 |  | 7-Jun | 548 |
|  | 8-Sep | 48 |  | 6-Oct | 120 |
| 2009 | 25-May | 204 |  | 23-Oct | 147 |
|  | 26-May | 3536 | 2017 | 26-May | 386 |
|  | 28-May | 688 |  | 27-May | 114 |
|  | 2-Jun | 70 |  | 2-Sep | 46 |
|  | 18-Sep | 112 | 2018 | 4-Sep | 52 |
|  | 21-Sep | 185 |  | 25-Sep | 60 |

**Supplementary file 1b.** Collection information for sample in the study.

|  | Code | Collection location |  | Longitude(E) | Latitude(N) | Host plants | Collection date | Specimens | | |
| --- | --- | --- | --- | --- | --- | --- | --- | --- | --- | --- |
|  |  |  |  |  |  |  |  | Isotope analysis | Population genetics | Feeding trails |
| 1 | PE | Yunnan Province, Puer | SW | 100.972343 | 22.777323 | watermelon | 2018.04 | 36 | 30 | 30 |
| 2 | NC | Jiangxi Province, Nanchang | YzP | 115.95046 | 28.551604 | cotton | 2017.09 | 36 | 30 | 30 |
| 3 | WH | Hubei Province, Wuhan |  | 114.02919 | 30.58203 | cotton | 2017.09 | 36 | 40 | 30 |
| 4 | TZ | Jiangsu Province, Taizhou |  | 119.88116 | 32.31841 | wheat | 2018.04 | 36 | 35 | 30 |
| 5 | YZ | Jiangsu Province, Yangzhou |  | 119.43157 | 32.39463 | wheat | 2018.04 | 36 | 35 | 30 |
| 6 | YL | Shannxi Province, Yangling | NP | 108.08455 | 34.27221 | orchard | 2018.07 | 36 | 35 | 30 |
| 7 | XX | Henan Province, Xinxiang |  | 113.90598 | 35.3718 | wheat | 2017.05 | 36 | - | 30 |
| 8 |  |  |  |  |  | cotton | 2017.09 | 36 | 50 | 30 |
| 9 | XJ | Shangdong Province, Xiajin |  | 116.00175 | 36.94856 | cotton | 2017.09 | 36 | 30 | 30 |
| 10 | DY | Shangdong Province, Dongying |  | 118.63019 | 37.30696 | cotton | 2017.09 | - | - | 30 |
| 11 | YT | Shangdong Province, Yantai |  | 121.112409 | 35.3522 | cotton | 2017.09 | 36 | 30 | 30 |
| 12 | CZ | Hebei Province, Cangzhou |  | 116.67828 | 38.23891 | wheat | 2018.04 | 36 | - | 30 |
| 13 | YP | Shanxi Province, Yuanping |  | 112.711104 | 38.730472 | wheat | 2018.05 | - | 20 | 30 |
| 14 | LF | Hebei Province, Langfang |  | 116.68572 | 39.50311 | wheat | 2017.05 | - | - | 15 |
| 15 |  |  |  |  |  | cotton | 2017.09 | 18 | 30 | 15 |
| 16 | CF | Neimenggu Province, Chifeng | NE | 118.95927 | 42.26581 | cotton | 2017.09 | - | 30 | 30 |
| 17 | SY | Liaoning Province, Shenyang |  | 123.589285 | 41.846472 | cotton | 2017.09 | 32 | 50 | 30 |
| 18 | CC | Jilin Province, Changchun |  | 125.332429 | 43.829776 | cotton | 2017.09 | 41 | 30 | 30 |
| 19 | HB | Heilongjiang Province, Harbin |  | 126.542051 | 45.816446 | cotton | 2017.09 | 36 | 30 | 30 |
| 20 | SH | Xinjiang Province, Shihezi | NW | 86.07893 | 44.30653 | alfalfa | 2017.07 | - | 24 | 20 |
| 21 | CDI | Changdao, Shandong | CD | 125.717623 | 39.059202 | - | (2014-2018)spring | 149 | 55 | 85 |
| 22 | CDII | Changdao, Shandong | CD | 125.717623 | 39.059202 | - | (2014-2018)Autumn | 149 | 55 | 85 |

**Supplementary file 1c.** Pollen carrying rate of the migratory *E. balteatus* hoverflies across Bohai Sea during 2014-2018.

| Sampling time | | No. with pollen | No. adults examined | % with pollen | No. taxa |
| --- | --- | --- | --- | --- | --- |
| Spring-summer migration stage | April | 7 | 42 | 17% | 28 |
|  | May | 101 | 367 | 28% |  |
|  | June | 59 | 174 | 34% |  |
|  | July | 1 | 29 | 3% | 1 |
| Autumn migration stage | August | 40 | 138 | 24% | 17 |
|  | September | 82 | 205 | 27% |  |
|  | October | 30 | 59 | 50% |  |
|  | Total | 320 | 1014 | 27% | 46 |

**Supplementary file 1d.** Quantitative PCR (qPCR) primers and conditions used in this study.

|  | Primer | Primer sequence (5'to3') |
| --- | --- | --- |
| *Cannabis sativa* | ITS2-F | TAGGCCAACCACAAGGCAAT |
|  | ITS2-R | CACTGCCAAAAGCGTGTTCA |
|  | ITS2-probe FAM | ATGGGAAGCCAGTCTCCGCC |
| *Humulus scandens* | ITS2-F | GCACCGATACAATCGAAAAC |
|  | ITS2-R | TGGCCTAAATTCGAGTCATC |
|  | ITS2-probe FAM | ACCGAATGTCGCGGCGATCG |
| *Helianthus annuus* | ITS2-F | GAGCATCTACTCTCAAGAAA |
|  | ITS2-R | CTAGTGGTGGTTGATAAGAC |
|  | ITS2-probe FAM | TAAACGCACGACACGAGACG |

**Supplementary file 1e.** Key parameters within *E. balteatus* searchlight trapping on BH Island during 2003–2018.

| **Year** | **First capture dd/mm (no.)** | **Final capture dd/mm (no.)** | **Duration of capture (d)** | **Peak capture dd/mm (no.)** |
| --- | --- | --- | --- | --- |
|  |  |  |  |  |
| 2003 | 01 Jun. (10) | 21 Sep. (4) | 113 | 07 Jun. (136) |
| 2004 | 14 May (10) | 18 Oct. (8) | 158 | 15 May (350) |
| 2005 | 13 May (3) | 04 Oct. (2) | 145 | 07 Sep. (270) |
| 2006 | 17 May (16) | 20 Oct. (14) | 157 | 03 Jun. (265) |
| 2007 | 02 May (2) | 11 Oct. (1) | 163 | 01 Sep. (185) |
| 2008 | 17 May (2) | 29 Sep. (3) | 136 | 24 May (248) |
| 2009 | 05 May (2) | 29 Sep. (37) | 148 | 26 May (3536) |
| 2010 | 21 May (1) | 13 Oct. (2) | 146 | 02 Sep. (1431) |
| 2011 | 03 May (1) | 21 Sep. (11) | 142 | 23 Aug. (389) |
| 2012 | 02 May (1) | 20 Oct. (1) | 172 | 05 Jun. (330) |
| 2013 | 02 May (7) | 10 Oct. (5) | 162 | 03 Jun. (129) |
| 2014 | 01 May (13) | 28 Sep. (1) | 151 | 21 May (1018) |
| 2015 | 28 Apr.(2) | 17 Oct. (1) | 173 | 24 Aug. (304) |
| 2016 | 30 Apr.(2) | 28 Oct. (1) | 182 | 07 Jun. (548) |
| 2017 | 23 May (1) | 22 Sep. (7) | 123 | 26 May (386) |
| 2018 | 04 May (1) | 28 Sep. (10) | 148 | 25 Sep. (60) |

**Supplementary file 1f.** The percentage of the total trajectories that ended in each region.

|  | Region | Valid endpoints | Total valid endpoints | Percentage |
| --- | --- | --- | --- | --- |
| Apr.-Jul. | Shandong | 7546 | 9069 | 83.20652773 |
|  | Liaoning | 966 | 9069 | 10.65167053 |
|  | Jiangsu | 74 | 9069 | 0.815966479 |
|  | Hebei | 68 | 9069 | 0.749807035 |
| Aug.-Oct. | Liaoning | 3945 | 4945 | 79.77755308 |
|  | Hebei | 461 | 4945 | 9.322548028 |
|  | Shandong | 204 | 4945 | 4.125379171 |
|  | Tianjin | 62 | 4945 | 1.253791709 |

**Supplementary file 1g.** Results of analysis of molecular variance (AMOVA) test in different populations and regions of *E.balteatus* based on Cytb and18S-28S rRNA gene.

|  | Source of variation | d.f. | Sum of squares | Variance components | Percentage of variation | Fixation Indices |
| --- | --- | --- | --- | --- | --- | --- |
| Cytb | Among populations | 17 | 16.8 | 0.012 Va | 1.84 | FST : 0.01844 P-value<0.02 |
|  | Within population | 512 | 327.117 | 0.6389 Vb | 98.16 |  |
|  | Total | 529 | 343.917 | 0.6509 |  |  |
| 18S-28S | Among populations | 15 | 217.7 | 0.60342 Va | 11.12 | FST : 0.11121 P-value<0.01 |
|  | Within population | 244 | 1176.661 | 4.82238 Vb | 88.88 |  |
|  | Total | 259 | 1394.362 | 5.42581 |  |  |

**Supplementary file 1h.** Comparative assessment of the degree of taxonomic identification obtained through either molecular or morphology-based approaches, for 46 different types of pollen grains dislocated from *E. balteatus* long-distance migrants collected on Beihuang Island (Bohai Sea, northeastern China). For each type of pollen grain, the highest level of taxonomic identification is indicated and contrasted between molecular and morphology-based approaches.

| **Pollen Grain Type** | **Identified Plants** | **Molecular Identification** |
| --- | --- | --- |
| 1 | *Ailanthus altissima* | *Ailanthus altissima* |
| 2 | *Cotinus coggygria* | *Cotinus coggygria* |
| 3 | *Forsythia suspensa* | *Forsythia suspensa* |
| 4 | *Prunus avium* | *Prunus avium* |
| 5 | Brassica L. | Sister to Brassica carinata/Brassica juncea/Brassica nigra |
| 6 | *Morus alba* | *Morus alba* |
| 7 | *Citrus sinensis* | *Citrus sinensis/Citrus maxima* |
| 8 | *Descurainia sophia* | *Descurainia sophia* |
| 9 | Euonymus L. | Sister to Euonymus alatus |
| 10 | Taraxacum L. | Sister to Taraxacum mongolicum |
| 11 | *Sedum japonicum* | *Sedum japonicum* |
| 12 | *Populus cathayana* | *Populus cathayana* |
| 13 | *Celastrus orbiculatus* | *Celastrus orbiculatus* |
| 14 | *Daucus carota* | *Daucus carota* |
| 15 | *Chenopodium album* | *Chenopodium giganteum/Chenopodium album* |
| 16 | *Castanea mollissima* | *Castanea mollissima* |
| 17 | *Amorpha fruticosa* | *Amorpha nana* |
| 18 | *Diospyros lotus* | *Diospyros lotus* |
| 19 | *Ziziphus jujuba* | *Ziziphus jujuba* |
| 20 | *Cirsium setosum* | *Cirsium setosum* |
| 21 | *Neoshirakia japonica* | *Neoshirakia japonica* |
| 22 | Flueggea L. | *Flueggea virosa* |
| 23 | *Maclura pomifera* | *Maclura pomifera* |
| 24 | Rumex L. | Sister to Rumex nepalensis/Rumex kandavanicus/Rumex ponticus |
| 25 | Euonymus L. | Sister to Euonymus myrianthus |
| 26 | *Schisandra chinensis* | *Schisandra chinensis* |
| 27 | *Eleusine indica* | *Eleusine indica* |
| 28 | *Actinidia kolomikta* | *Actinidia kolomikta* |
| 29 | *Cannabis sativa* | *Cannabis sativa* |
| 30 | *Humulus scandens* | *Humulus scandens* |
| 31 | *Helianthus annuus* | *Helianthus annuus* |
| 32 | *Persicaria orientalis* | *Persicaria orientalis* |
| 33 | *Adenophora trachelioides* | *Adenophora trachelioides* |
| 34 | *Gypsophila paniculata* | *Gypsophila paniculata* |
| 35 | Artemisia L. | *Artemisia sp. AD-H* |
| 36 | *Rubia cordifolia* | *Rubia cordifolia* |
| 37 | *Suaeda glauca* | *Suaeda glauca* |
| 38 | Artemisia L. | Sister to Artemisia japonica |
| 39 | Artemisia L. | Sister to Artemisia sieversiana |
| 40 | Artemisia L. | Sister to Artemisia annua |
| 41 | *Allium tuberosum* | *Allium tuberosum* |
| 42 | *Tripolium vulgare* | *Tripolium vulgare* |
| 43 | *Ambrosia trifida* | *Ambrosia trifida* |
| 44 | *Sorghum bicolor* | *Sorghum bicolor* |
| 45 | *Aster tataricus* | *Aster tataricus* |
| 46 | *Chrysanthemum zawadskii* | *Chrysanthemum zawadskii* |
